# Relationship between pretracheal and/or prelaryngeal lymph node metastasis and paratracheal and lateral lymph node metastasis of papillary thyroid carcinoma: A meta-analysis

**DOI:** 10.3389/fonc.2022.950047

**Published:** 2022-09-23

**Authors:** Bin Wang, Chun-Rong Zhu, Hong Liu, Xin-Min Yao, Jian Wu

**Affiliations:** ^1^ Center of Breast and Thyroid Surgery, Department of General Surgery, Chengdu Third People’s Hospital, Chengdu, China; ^2^ Department of Oncology Ward 2, Chengdu Third People’s Hospital, Chengdu, China

**Keywords:** pretracheal lymph node, prelaryngeal lymph node, paratracheal lymph node, lateral lymph node, papillary thyroid carcinoma, meta-analysis

## Abstract

**Objective:**

We conducted a meta-analysis to study the relationship between pretracheal and/or prelaryngeal lymph node metastasis and paratracheal and lateral lymph node metastasis in papillary thyroid carcinoma.

**Method:**

A systematic literature search was conducted using PubMed, Embase, and the Cochrane Library electronic databases for studies published up to February 2022. The reference lists of retrieved articles were also reviewed. Two authors independently assessed the methodological quality and extracted the data. A random-effects model was used to calculate the overall pooled relative risk. Publication bias in these studies was evaluated using Egger’s test and Begg’s test.

**Results:**

Twenty-five independent studies involving 10,525 patients were included in the meta-analysis. The pooled relative risk for ipsilateral and contralateral paratracheal lymph node metastasis was 3.01 (95% confidence interval [CI]: 1.66, 5.45) and 5.68 (95% CI: 2.50, 12.88), respectively, in patients with pretracheal lymph node metastasis. Among patients with prelaryngeal lymph node metastasis, the pooled relative risk for ipsilateral paratracheal and/or pretracheal contralateral paratracheal, and lateral lymph node metastasis was 2.02 (95% CI: 1.90, 2.14), 2.22 (95% CI: 1.34, 3.67), and 3.85 (95% CI: 2.89, 5.14), respectively.

**Conclusion:**

Pretracheal lymph node metastasis and prelaryngeal lymph node metastasis were significantly associated with an increased likelihood of both ipsilateral lymph node metastasis and contralateral paratracheal lymph node metastasis in papillary thyroid carcinoma. Prelaryngeal lymph node metastasis was positively correlated with the incidence of lateral lymph node metastasis.

## Introduction

Lymph node metastasis is the most frequent form of metastasis of papillary thyroid carcinoma (PTC), which occurred in 37–61% of patients with clinically negative lymph node (cN0) (1–3). However, there is no consensus on prophylactic central lymph node dissection for low-risk PTC. Routine ipsilateral central lymph node dissection is recommended to be performed for patients with PTC in Chinese Guidelines on the Diagnosis and Treatment of Thyroid Nodules and Differentiated Thyroid Carcinomas (4). The Guidelines for the Management of Thyroid Cancer of the British Thyroid Association indicate that Personalized Decision Making is recommended for patients with non-high-risk PTC with clinically/radiologically uninvolved neck nodes (5). The European Society for Medical Oncology Clinical Practice Guidelines for Diagnosis, Treatment, and Follow-up of Thyroid Cancer indicate that the use of prophylactic central neck dissection for low-risk tumors (T1b–T2, N0) varies across centers (6). However, according to the 2015 American Thyroid Association Management Guidelines for Adult Patients with Thyroid Nodules and Differentiated Thyroid Cancer, prophylactic central neck dissection is not recommended for small (T1 or T2), noninvasive, and clinically node-negative PTC (7). As a result, the indicators of prophylactic central lymph node dissection for low-risk PTC remain unclear.

In recent decades, it has been explored whether the metastasis of the prelaryngeal lymph node, also known as the Delphian lymph node, could be used to predict metastasis in other groups’ lymph node, based on its anatomical location. A previous meta-analysis suggested that the risks of central lymph node metastasis and lateral lymph node metastasis were both significantly higher in the prelaryngeal lymph node metastasis group than that in the negative prelaryngeal lymph node group (8). Kim and colleagues confirmed that the specificities of prelaryngeal lymph node metastasis in predicting metastasis in other group lymph nodes were high (9). Because it is located at a similar site, the association between metastasis to the pretracheal lymph node and metastasis to other groups’ lymph node has also been investigated previously (10–12).

In order to explore the significance of the combination of prelaryngeal lymph node metastasis and pretracheal lymph node metastasis for predicting metastasis to other groups’ lymph node, we conducted this meta-analysis to assess the association of pretracheal and/or prelaryngeal lymph node metastasis with paratracheal and lateral lymph node metastasis in PTC.

## Methods

### Literature search

A search was independently conducted by two investigators on PubMed, Embase, and the Cochrane Library electronic databases for studies that were published up to 28 February 2022. The search algorithm was ([pretracheal lymph node] or [prelaryngeal lymph node] or [delphian lymph node] or [pre-tracheal lymph node] or [pre-laryngeal lymph node]) AND [thyroid]) for PubMed. The following search terms were used in all fields as a search strategy for Embase (1): pretracheal and pre-tracheal; prelaryngeal and pre-laryngeal; delphian; (2) lymph node, lymph nodes, and (lymph node); (3) thyroid gland, thyroid tumor, thyroid cancer, thyroid neoplasm, thyroid neoplasms, thyroid, thyorids, thyroidal, and thyroideal. For Cochrane Library electronic databases, the search strategy used the following terms as Medical Subject Headings and free word in all fields: (1) pretracheal and pre-tracheal; prelaryngeal and pre-laryngeal; delphian; (2) lymph node, lymph nodes, and (lymph node); (3) thyroid gland, thyroid neoplasms; thyroid, thyorids, thyroidal, and thyroideal. No restrictions were imposed. In addition, we reviewed the reference lists of the retrieved papers and recent reviews.

### Study selection

After removing duplication, the acquired studies were screened based on the title and abstract, and the full text was then reviewed. Studies were considered eligible, if they met all the following criteria: (1) the original study was published in English; (2) patients underwent initial thyroid surgery for PTC in the study; (3) the exposure of interest included prelaryngeal and/or pretracheal lymph node metastasis; (4) the outcome of interest was other groups’ lymph node metastasis and/or lateral lymph node metastasis; and (5) relative risk (RR) and the corresponding 95% confidence interval (CI) (or data to calculate these values) were available. Studies were excluded based on the following criteria: (1) Conference Abstract, Review, Case report, and Commentary; (2) those from which data could not be collected adequately.

### Data extraction and quality assessment

Two reviewers (BW and C-RZ) independently extracted data using a predefined data extraction form. Data were collected as follows: publication date, first author, type of study, country of origin, study sites and institutes, research period, sample size, preoperative clinical lymph node stage, tumor location, the maximum diameter of tumor, and surgical method, whether patients without prelaryngeal lymph node were included, the number of patients with or without prelaryngeal and/or pretracheal lymph node metastasis, and the number of patients with or without metastasis in other groups’ lymph node. The quality of cohort studies was assessed using the Newcastle–Ottawa Scale (NOS), and studies with a NOS score > 5 were considered high-quality studies (13). All disagreements in study selection, data extraction, and quality assessment were discussed and resolved by consensus.

### Statistical analysis

Pooled RRs were calculated using a random-effects model (DerSimonian–Laird). Heterogeneity was quantified statistically with the *I* ^2^ test. *P* < 0.1 and *I* ^2^ > 50% for heterogeneity was considered significant difference. If there was significant heterogeneity, subgroup analysis was conducted according to the tumor location, or the state of the prelaryngeal lymph node, or the surgical method. Potential publication bias was assessed using Begg’s rank correlation test and the Egger linear regression test (14, 15). All analyses were performed using Stata version 14.0 (Stata Corp LP, College Station, TX, USA). Statistical significance was set at *P* < 0.05.

## Results

### Literature search

**Figure 1:**
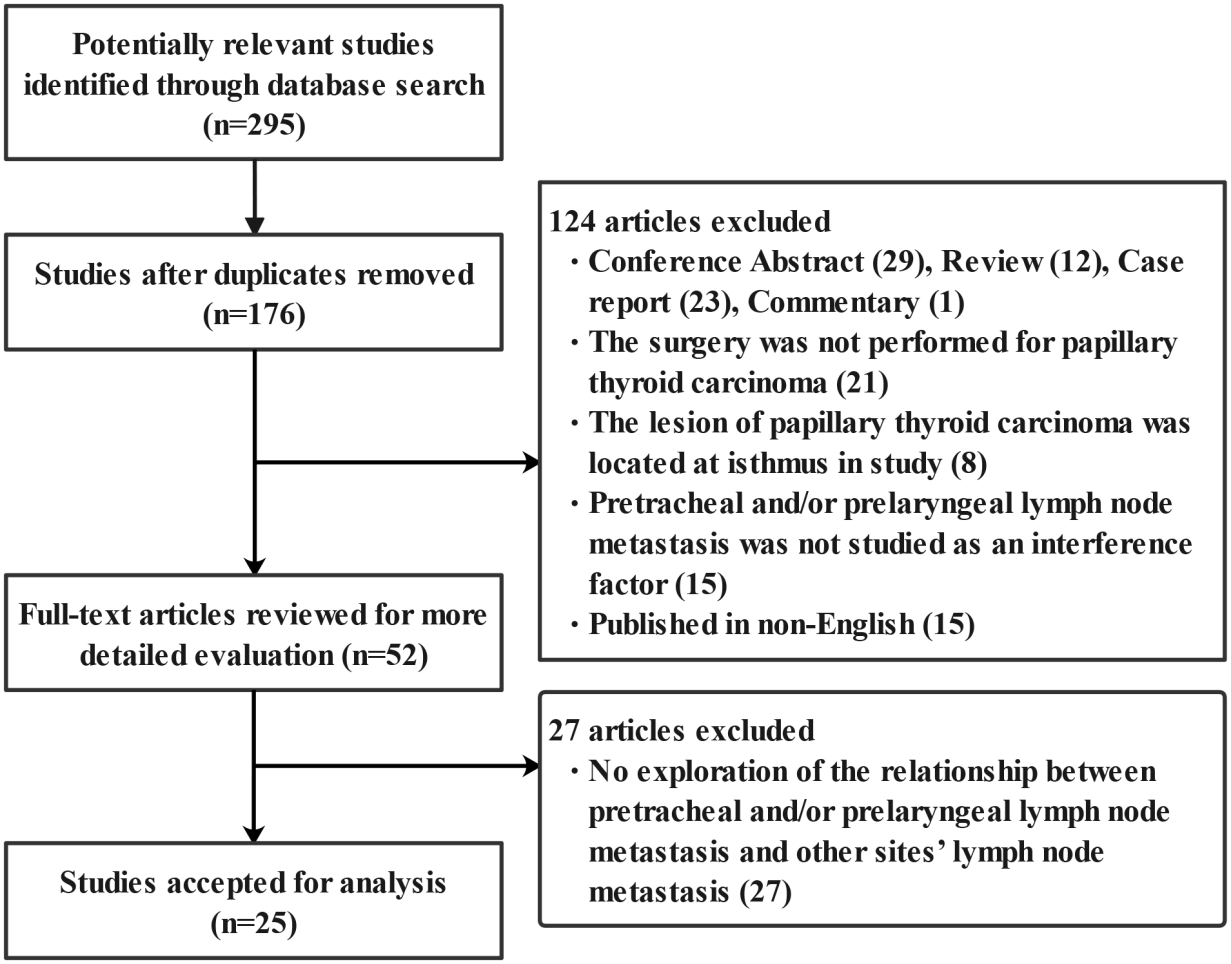
Flow chart of study selection.

The study selection process is illustrated in **Figure 1**. A total of 295 potentially relevant records were identified by searching these databases. Of these, 176 were retained after duplicates were removed. After the first screening, 124 studies were excluded for various reasons. The remaining 52 studies were assessed *via* full-text screening, and 27 studies were further excluded. Finally, 25 independent studies were included in the meta-analysis.

### Study characteristics

**Table 1** shows the basic information of the 25 eligible studies (10–12, 16–37). These studies were published between 2011 and 2022. Among the 25 cohort studies, 8 were prospective and 17 were retrospective studies. One, five, and nineteen studies were conducted in America, Korea, and China, respectively. The sample size of these studies varied from 67 to 1,575, and a total of 10,525 patients were included in the analysis. The exposure of interest consisted of prelaryngeal lymph node metastasis, pretracheal lymph node metastasis, and prelaryngeal and/or pretracheal lymph node metastasis. The outcome of interest varied across studies, including ipsilateral paratracheal lymph node metastasis, contralateral paratracheal lymph node metastasis, pretracheal lymph node metastasis, central without prelaryngeal lymph node metastasis, ipsilateral paratracheal and/or pretracheal lymph node metastasis, and lateral lymph node metastasis. According to the NOS score, all included studies were of relatively high quality, with the distribution of the scores ranging from 6 to 8.

**Table 1:** The characteristics of included studies.

Study ID	Publication Date	First Author	Type of study	Country/Region	Institute	Research period	Patients (*n*)	Clinical Stage	Bilateral Lesions	Isthmic Lesion	Existence of Prelaryngeal Lymph Node	Maximum Diameter of Lesion (cm)	Surgical Method	Interference Factors and Outcome Factors		
														PT	PL	PT and/or PL
**1**	2011	Iyer	Retrospective	American	Memorial Sloan-Kettering Cancer Center	2007.1—2009.12	101	—	NK	NK	NK	—	TT+rPL-LND, eCND, tLLND		Central without PL, Lateral	
**2**	2011	Roh	Prospective	Korea	Asan Medical Center	2005—2007	184	cN0, unilateral	Accidental Discovery	N	NK	1.5 ± 1.2 (0.4–5.5)	TT+BCND	Ipa, Cpa		Ipa
**3**	2012	Kim	Retrospective	South Korea	Kosin University College of Medicine	2010.1—2010.10	308	—	NK	NK	N	1.1 ± 0.7 (0.05 – 5.8)	LT/TT+CND ± MRND			Ipa, Cpa, Lateral
**4**	2013	Lee	Prospective	Korea	Kyung Hee University	—	67	cN0	Y	Y	NK	0.3-5.5	TT+pBCND	Ipa,	Ipa, Cpa, Lateral, PT	Ipa, Cpa
**5**	2013	Miao	Prospective	China	Third Affiliated Hospital of Harbin Medical University	2007.7—2009.8	184	cN0, unilateral	N	N	NK	0.2-6.8	TT+pBCND	Ipa, Cpa	Ipa, PT	
**6**	2013	Oh	Retrospective	Korea	Gachon University Gil Hospital	2009.7—2011.12	245	—	Y	NK	N	–	TT+t/pBCND+tLLND		Central without PL, Lateral	
**7**	2014	Eun	Prospective	Korea	Multitertiary centers: Kyung Hee University School of Medicine, Kyung Hee University School of Medicine at Gangdong, Kangdong Sacred Heart Hospital	—	140	cN0, unilateral	Accidental Discovery	Y	NK	1.4 ± 1.2	TT+pBCND	Cpa		
**8**	2015	Chen	Retrospective	China	West China Hospital	2011.9—2013.10	218	cN0, unilateral	N	N	Y	≤4.0	TT+pBCND	Cpa	Cpa	
**9**	2015	Wei	Retrospective	China	West China Hospital	2008.6—2011.6	332	unilateral	N	N	NK	—	NTT/TT+BCND+tLLND			Cpa
**10**	2017	Chen	Prospective	China	West China Hospital	2011.7—2013.5	153	cN0, unilateral	N	N	Y	>1	TT+pBCND	Cpa	Cpa	Cpa
**11**	2017	Tan	Retrospective	China	Zhejiang Cancer Hospital	2013.1—2014.6	231	—	Y	NK	Y	—	LT/NTT/TT+ICND/BCND+tLLND		Central without PL, Lateral	
**12**	2017	Zheng	Retrospective	China	The Affiliated Yantai Yuhuangding Hospital of Qingdao University	2014.8—2016.5	206	—	Y	Y	N	—	STT/NTT/TT+ICND/BCND+tLLND		Ipa+Cpa, Lateral, PT	
**13**	2019	Wang	Retrospective	China	Changzheng Hospital	2017.7—2018.8	192	—	Y	Y	NK	—	TT+ICND+tCCND+LLND		Central without PL, Lateral	
**14**	2020	Chen	Retrospective	China	West China Hospital	—	1085	unilateral	N	N	NK	—	TT+BCND+tLLND	Cpa	Cpa	
**15**	2020	Gong	Retrospective	China	Kunshan Hospital	2014—2018	254	—	N	Y	Y	—	LT/TT+tCND		Ipa+Cpa	
**16**	2020	He	Retrospective	China	The First Affiliated Hospital, School of Medicine, Zhejiang University	2018.7—2019.3	622	—	Y	Y	NK	—	NK		Central without PL, Lateral	
**17**	2020	Liu	Prospective	China	Qilu Hospital of Shandong University	2017.1—2019.3	237	cT1N1a/cT2N1a, unilateral	N	N	NK	1.4 ± 0.8 (0.6~4.0)	TT+BCND	Cpa	Cpa	
**18**	2020	Zhou	Prospective	China	Qilu Hospital of Shandong University	2017.5—2019.10	242	cN0, unilateral	N	NK	NK	—	TT+BCND			Cpa
**19**	2021	Li	Retrospective	China	Tianjin Cancer Hospital	2017.6—2019.1	581	—	Y	NK	N	—	LT/TT+ICND+tCPa-CND+tLLND		Central without PL, Lateral	
**20**	2021	Qi	Retrospective	China	First Affiliated Hospital of Nanchang University	2017.2—2021.6	485	—	Y	Y	N	—	LT/TT+I/BCND+tLLND		Central without PL, Lateral	
**21**	2021	Yan	Retrospective	China	Tianjin Medical University Cancer Institute and Hospital	2017.8—2020.6	516	—	Y	Y	N	—	LT/TT+I/BCND+tLLND		Central without PL, Lateral	
**22**	2021	Zhu	Retrospective	China	First Affiliated Hospital of Chongqing Medical University	2013.7—2018.12	1575	—	Y	Y	N	—	LT/TT+ICND+p/tLLND		PT+Ipa, Lateral	
**23**	2021	Zhu	Retrospective	China	First Affiliated Hospital of Chongqing Medical University	2016.12—2018.12	1271	cT1~2 N0	Y	Y	NK	≤4	LT/TT+ICND+p/tLLND		PT+Ipa	
**24**	2021	Zhu	Retrospective	China	First Affiliated Hospital of Fujian Medical University	2014.1—2015.12	904	—	NK	Y	Y	—	TT/NTT+I/BCND+tLND		PT+Pa, Latreal	
**25**	2022	Guo	Prospective	China	Fourth Affiliated Hospital of Anhui Medical University	2018.8—2020.2	192	—	NK	NK	NK	—	NK		Central without PL	

NK, not knowledge; LT, lobe thyroidectomy; TT, total thyroidectomy; NTT, near total thyroidectomy; rPL-LND, routinely prelaryngeal lymph node dissection; CND, central lymph node dissection; BCND, bilateral CND; eCND, elective CND; pBCND, prophylactic BCND; tBCND theprapetic BCND; ICND ipisilateral CND; tCCND therapeutic contralateral CND; LLND, lateral lymph node dissection; tLLND, therapeutic LLND; pLLND, prophylactic LLND; MRND modified radical lymph node dissection; PT, pretracheal lymph node; PL, prelaryngeal lymph node; Ipa, ipsilateral paratracheal lymph node; Cpa, contralateral paratracheal lymph node; central, central lymph node; lateral, lateral lymph node.

### Relationship between pretracheal lymph node metastasis and metastasis to other groups’ lymph nodes

**Figure 2:**
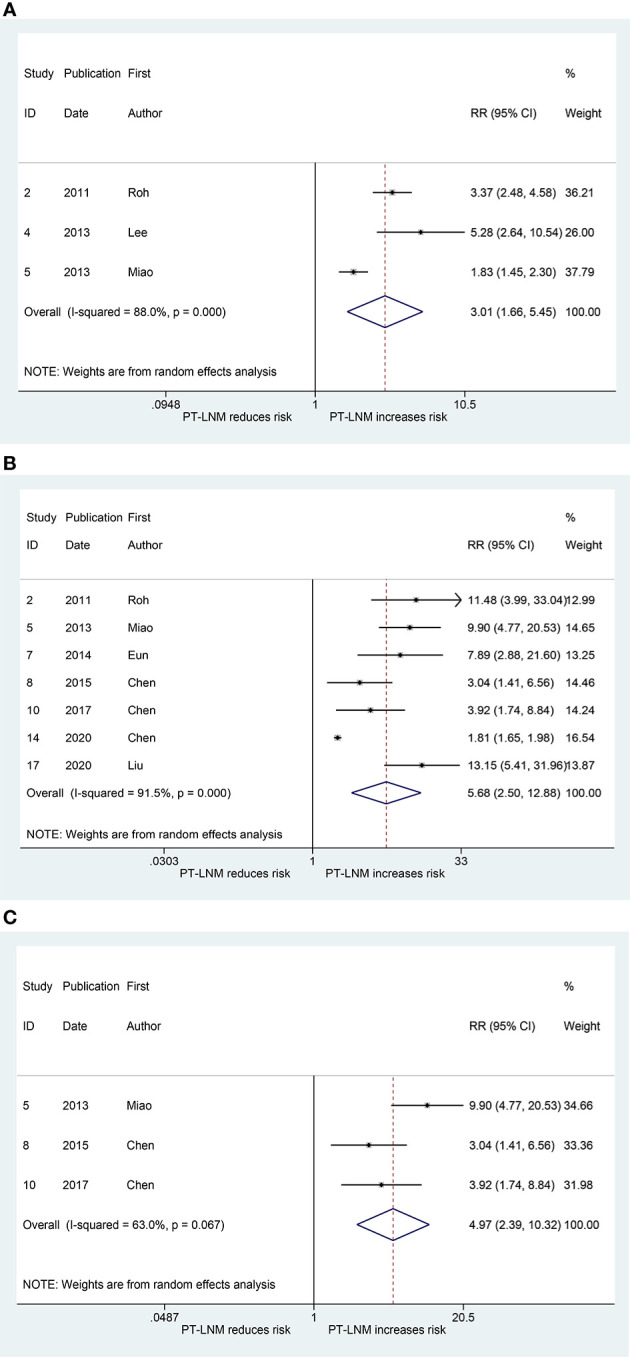
The relative risk for other groups’ lymph node metastasis in patients with pretracheal lymph node metastasis (PT-LNM). **(A)** Ipsilateral paratracheal lymph node metastasis. **(B)** Contralateral paratracheal lymph node metastasis. **(C)** Contralateral paratracheal lymph node metastasis in patients with unilateral papillary thyroid carcinoma and clinical negative nodes.

Three studies explored the relationship between pretracheal lymph node metastasis and ipsilateral paratracheal lymph node metastasis (17, 19, 20). The pooled RR was 3.01 (95% CI: 1.66, 5.45, *p* < 0.001; **Figure 2A**). Significant heterogeneity was observed (*I* ^2^ = 88.0%, *p* < 0.001; **Figure 2A**). The publication bias, as measured by Begg’s test and Egger’s test, was not significant (*p* > 0.99, *p* = 0.393, respectively). The relationship between pretracheal lymph node metastasis and contralateral paratracheal lymph node metastasis was investigated in seven studies (10–12, 17, 20, 22, 23), and the pooled RR was 5.68 (95% CI: 2.50, 12.88, *p* < 0.001; **Figure 2B**). Here, heterogeneity was also significant (*I* ^2^ = 91.5%, *p* < 0.001; **Figure 2B**). Begg’s test confirmed that publication bias was not significant (*p* = 0.368), whereas Egger’s test yielded the opposite result (*p* = 0.003). When analysis was performed in patients with cN0 unilateral PTC, the pooled RR was 4.97 (95% CI: 2.39, 10.32, *p* < 0.001; *I* ^2^ = 63.0%, *p* = 0.067; **Figure 2C**), and the publication bias was not significant (Begg, *p* > 0.99; Egger, *p* = 0.464).

### Relationship between prelaryngeal lymph node metastasis and metastasis to other groups’ lymph nodes

**Figure 3:**
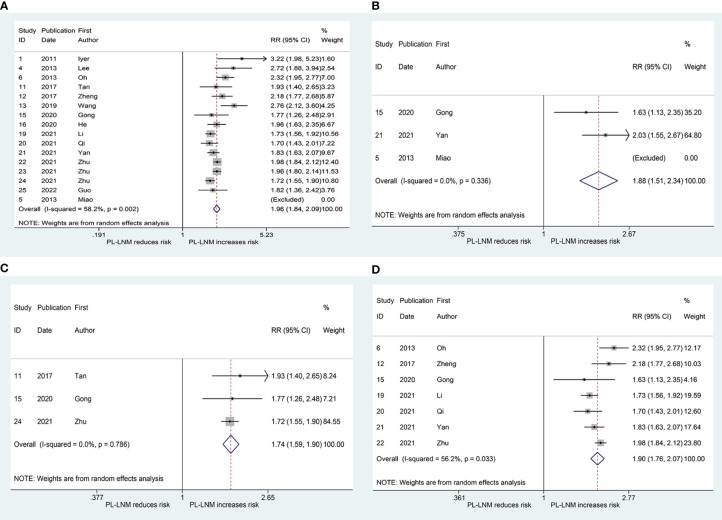
The relative risk for central (without prelaryngeal) lymph node metastasis in patients with prelaryngeal lymph node metastasis (PL-LNM). **(A)** The overall. **(B)** Subgroup of non-bilateral papillary thyroid carcinoma. **(C)** Subgroup of inclusion of cases without prelaryngeal lymph node. **(D)** Subgroup of exclusion of cases without prelaryngeal lymph node.

**Figure 3A** shows the results of the pooled RR for central (without prelaryngeal) lymph node metastasis. Sixteen studies were included in the analysis (16, 19–21, 25–29, 31–37). The RRs for the relationship between prelaryngeal lymph node metastasis and central (without prelaryngeal) lymph node metastasis varied from 1.70 to 3.22 across these studies, whereas the pooled RR was 1.96 (95% CI: 1.84, 2.09, *p* < 0.001). Heterogeneity was significant (*I* ^2^ = 57.2%, *p* = 0.002), but publication bias was not significant (Begg, *p* = 0.166; Egger, *p* = 0.13). To address the heterogeneity, subgroup analyses were performed for non-bilateral PTC and inclusion or exclusion of cases without prelaryngeal lymph node. The pooled RR for central (without prelaryngeal) lymph node metastasis was 1.88 (95% CI: 1.51, 2.34, *p* < 0.001; *I* ^2^ = 0.0%, *p* = 0.336, **Figure 3B**; Begg, *p* > 0.99; Egger, *p* = -) in the subgroup of non-bilateral PTC. Additionally, in the subgroup of inclusion and exclusion of cases without prelaryngeal lymph node, the pooled RR was 1.90 (95% CI: 1.76, 2.07, *p* < 0.001; *I* ^2 =^ 56.2%, *p* = 0.033; **Figure 3C**; Begg, *p* > 0.99; Egger, *p* = 0.924) and 1.74 (95% CI: 1.59, 1.90, *p* < 0.001; *I* ^2^ = 0.0%, *p* = 0.786; **Figure 3D**; Begg, *p* > 0.99; Egger, *p* = 0.394), respectively.

**Figure 4:**
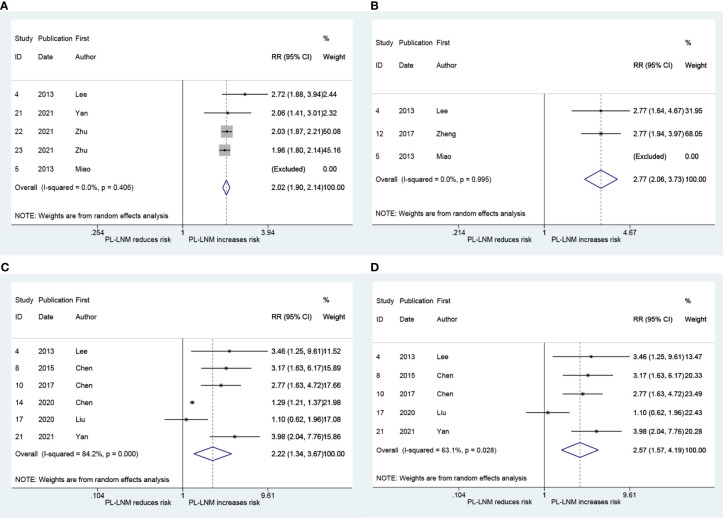
The relative risk for other groups’ lymph node metastasis in patients with prelaryngeal lymph node metastasis (PL-LNM). **(A)** Ipsilateral paratracheal and/or pretracheal lymph node metastasis. **(B)** Pretracheal lymph node metastasis. **(C)** Contralateral paratracheal lymph node metastasis. **(D)** Contralateral paratracheal lymph node metastasis in the subgroup of patients without lateral lymph node dissection.

The pooled RR for ipsilateral paratracheal and/or pretracheal lymph node metastasis is presented in **Figure 4A**. The risk of ipsilateral paratracheal and/or pretracheal lymph node metastasis for patients with prelaryngeal lymph node metastasis was higher than that for patients without prelaryngeal lymph node metastasis (RR = 2.02, 95% CI: 1.90, 2.14, *p* < 0.001; *I* ^2^ = 0.0%, *p* = 0.406; **Figure 4A**; Begg, *p* > 0.99; Egger, *p* = 0.294). The pooled RR for pretracheal lymph node metastasis was 2.77 (95% CI: 2.06, 3.73, *p* < 0.001; *I* ^2^ = 0.0%, *p* = 0.995; **Figure 4B**; Begg, *p* > 0.99; Egger, *p* = -). These patients with prelaryngeal lymph node metastasis were also prone to suffer from contralateral paratracheal lymph node metastasis (RR = 2.22, 95% CI: 1.34, 3.67, *p* < 0.001; *I* ^2 =^ 84.2%, *p* = 0.002; **Figure 4C**; Begg, *p* = 0.452; Egger, *p* = 0.043). Due to heterogeneity and publication bias, subgroup analysis was performed in patients without lateral lymph node dissection. The pooled RR for contralateral paratracheal lymph node metastasis was 2.57 (95% CI: 1.57, 4.19, *p* < 0.001; *I* ^2 =^ 63.1%, *p* = 0.028; **Figure 4D**; Begg, *p* = 0.462; Egger, *p* = 0.531) in this subgroup.

**Figure 5:**
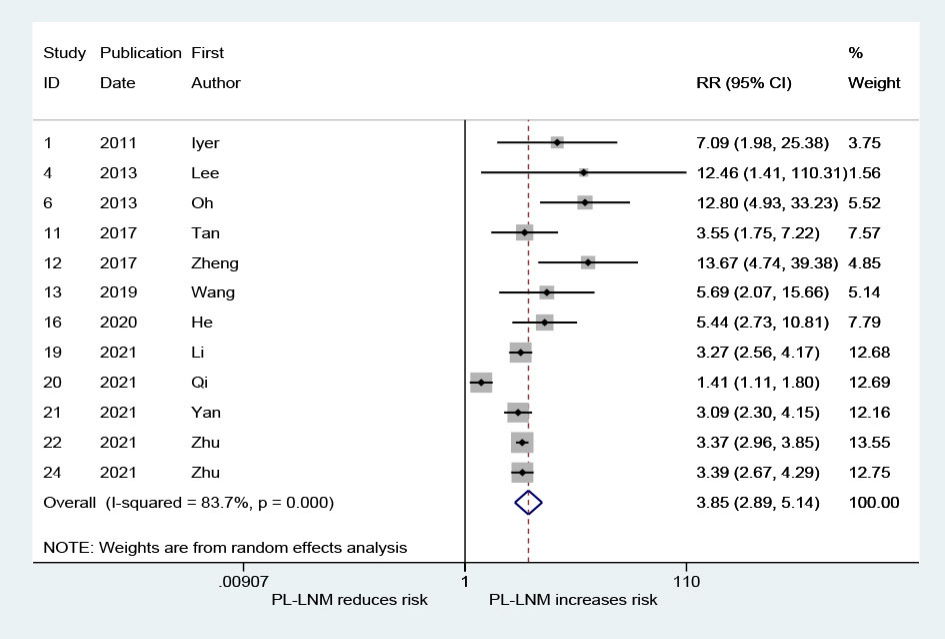
The relative risk for lateral lymph node metastasis in patients with prelaryngeal lymph node metastasis (PL-LNM).

As shown in **Figure 5**, the pooled RR for lateral lymph node metastasis was 3.85 (95% CI: 2.89, 5.14, *p* < 0.001). Although heterogeneity was significant, the lower CIs of all RRs exceeded 1 (*I* ^2 =^ 83.7%, *p* < 0.001; **Figure 5**). No significant publication bias was observed (Begg, *p* = 0.373; Egger, *p* = 0.167).

### Relationship between pretracheal and/or prelaryngeal lymph node metastasis and metastasis to other groups’ lymph nodes

**Figure 6:**
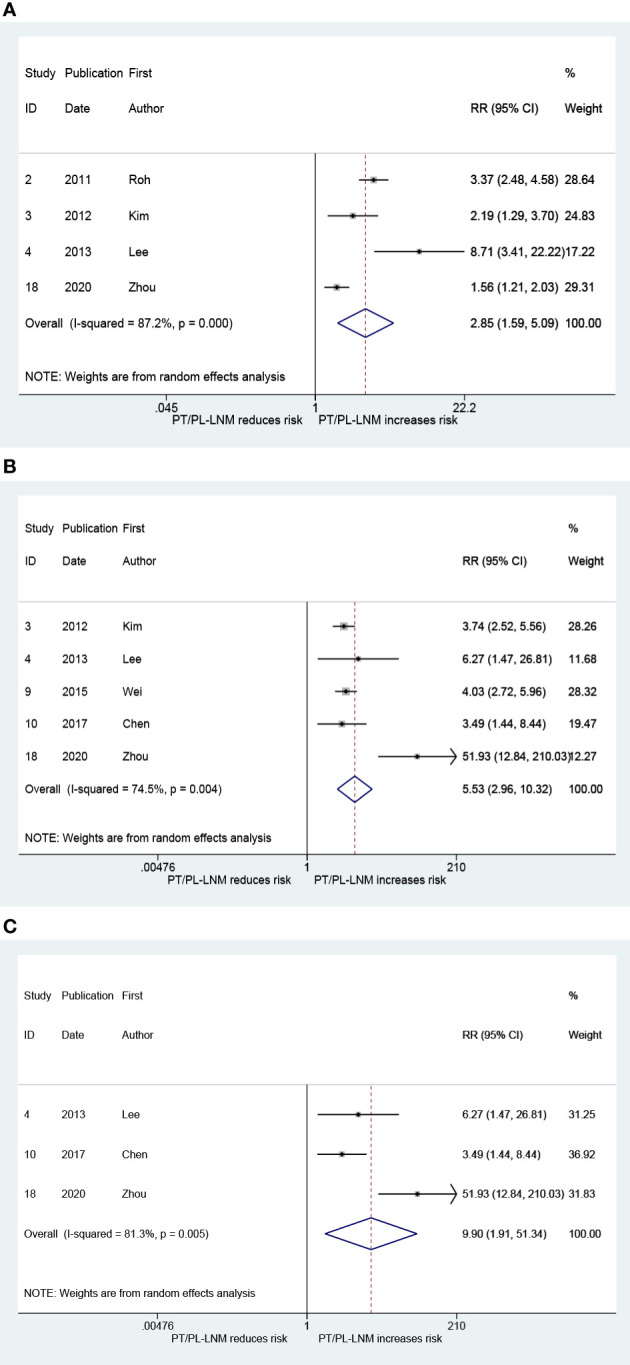
The relative risk for other groups’ lymph node metastasis in patients with pretracheal and/or prelaryngeal lymph node metastasis (PT/PL-LNM). **(A)** Ipsilateral paratracheal lymph node metastasis. **(B)** Contralateral paratracheal lymph node metastasis. **(C)** Contralateral paratracheal lymph node metastasis in the subgroup of patients without lateral lymph node dissection.

The result that is presented in **Figure 6A** combines the RRs for ipsilateral paratracheal lymph node metastasis in patients with pretracheal and/or prelaryngeal lymph node metastasis. The pooled RR was 2.85 (95% CI: 1.59, 5.09, *p* < 0.001; **Figure 6A**). Heterogeneity was significant (*I* ^2^ = 87.2%, *p* < 0.001; **Figure 6A**). There was no significant publication bias (Begg, *p* = 0.734; Egger, *p* = 0.376). **Figure 6B** presents the pooled RR related to the pretracheal and/or prelaryngeal lymph node metastasis and the risk of contralateral paratracheal lymph node metastasis; the risk of contralateral paratracheal lymph node metastasis was higher in cases with pretracheal and/or prelaryngeal lymph node metastasis (RR = 5.53, 95% CI: 2.96, 10.32, *p* < 0.001; *I* ^2 =^ 74.5%, *p* = 0.004; **Figure 6B**; Begg, *p* = 0.462; Egger, *p* = 0.215). Subgroup analysis was performed in patients who did not undergo lateral lymph node dissection. This confirmed that the risk of contralateral paratracheal lymph node metastasis was further increased in these patients (RR = 9.90, 95% CI: 1.91, 51.34, *p* = 0.006; *I* ^2 =^ 81.3%, *p* = 0.005, **Figure 6C**; Begg, *p* > 0.99; Egger, *p* = 0.486).

## Discussion

The present meta-analysis suggested that pretracheal lymph node metastasis was positively associated with both ipsilateral and contralateral paratracheal lymph node metastasis and that prelaryngeal lymph node metastasis might be a risk factor for central (without prelaryngeal) and lateral lymph node metastasis.

According to the American Thyroid Association Management Guidelines, thyroidectomy without prophylactic central neck dissection is appropriate for small (T1 or T2) and noninvasive clinically node-negative PTC (7). This recommendation was based on the lack of improvement in long-term patient outcomes and an increase in temporary morbidity in these cases (3, 38–40). Similarly, prophylactic central neck dissection for non-high-risk PTC is not routinely performed in Europe (5, 6). Due to their anatomical sites, prelaryngeal and pretracheal lymph nodes can be removed without an increase in complications. Therefore, metastasis to them might be used as an indicator to further screen high-risk PTC.

A recent study indicated that more than 50% of physicians thought that total thyroidectomy for low-risk PTC was not overused, whereas more than 60% of physicians believed that radioiodine for low-risk PTC was overused (41). It implies that, regarding low-risk PTC treatment, conditional total thyroidectomy was acceptable and that radioiodine should be used less frequently. Leboulleux et al. confirmed that the prognoses of nonuse of radioiodine was non-inferior to use of radioiodine in terms of the occurrence of functional, structural, and biologic events at 3 years for patients with pT1N0/x PTC undergoing thyroidectomy (42). Therefore, it might be a better strategy that further distinguishing patients who are able to benefit from radioiodine from patients with low-risk PTC, especially pT2 PTC. In this regard, prelaryngeal and/or pretracheal lymph node metastasis might be used as an indicator.

The thyroid gland has abundant intersecting lymph vessels. Therefore, lymphatic drainage of the thyroid gland is complex, which means that there is no precise sentinel lymph node for thyroid carcinoma. The central lymph node is usually defined as a perithyroidal lymph node, which consists of the prelaryngeal lymph node, pretracheal lymph node, and paratracheal (or trachea-esophageal groove) lymph node (43). However, there is no clear boundary between the pretracheal lymph node and paratracheal lymph node. The pretracheal and ipsilateral paratracheal lymph node metastasis might occur successively or simultaneously. Although it could not be confirmed whether lymph node metastasis occurred earlier in pretracheal or in ipsilateral paratracheal sites, the correlation of lymph node metastasis between the two sites might not be ignored according to our meta-analysis. Pretracheal lymph node metastasis might be considered as an indicator of prophylactic ipsilateral paratracheal lymph node dissection in low-risk PTC.

Contralateral paratracheal lymph node metastasis occurs in 0–21% of patients with unilateral low-risk PTC (12, 17, 20, 24, 30). The meta-analysis suggested that pretracheal lymph node metastasis was positively related to contralateral paratracheal lymph node metastasis after excluding patients with accidental bilateral lobe lesions, isthmic lesions, or preoperative clinical lymph node metastasis. The positive association between contralateral paratracheal lymph node metastasis and pretracheal lymph node metastasis might result from the anatomical site, where the pretracheal lymph node might be one of the stations for metastasis on the way to the contralateral paratracheal lymph node.

Enlightened by the evidence that prelaryngeal lymph node metastasis was a poor prognostic factor in laryngeal and hypopharyngeal cancers, the influence of which in PTC was studied (16, 44, 45). When the number of metastatic lymph nodes exceeds five, it was regarded as an intermediate risk for PTC, which meant that radioiodine adjuvant therapy should be considered (7). A previous meta-analysis showed that prelaryngeal lymph node metastasis was positively associated with central (without prelaryngeal) lymph node metastasis, but it did not examine the effects on subgroups (such as ipsilateral paratracheal, pretracheal and contralateral) of central lymph node metastasis (8). This meta-analysis confirmed that prelaryngeal lymph node metastasis might be a predictor of ipsilateral paratracheal and/or pretracheal lymph node metastasis, pretracheal lymph node metastasis, contralateral lymph node metastasis, and lateral lymph node metastasis. Some previous studies have confirmed that patients with PTC characterized by bilateral lesions, multiple lesions, extrathyroidal extension, lymphovascular invasion, and aggressive pathology are more likely to suffer from prelaryngeal lymph node metastasis (12, 24, 27). In other words, prelaryngeal lymph node metastasis might represent more aggressive invasiveness and be a poor prognostic factor in PTC.

The present meta-analysis found that the pooled RRs for ipsilateral and contralateral paratracheal lymph node metastasis in patients with pretracheal and/or prelaryngeal lymph node metastasis were both higher than that in patients with prelaryngeal lymph node metastasis and lower than that in patients with pretracheal lymph node metastasis. In consideration that lateral lymph node metastasis is a risk factor for contralateral paratracheal lymph node metastasis, patients with lateral lymph node dissection were excluded, after which the RR for contralateral paratracheal lymph node metastasis was higher. This phenomenon might suggest that the combination of prelaryngeal and pretracheal lymph node metastasis might be a more valuable predictor than prelaryngeal lymph node metastasis to predict paratracheal lymph node metastasis. Due to a lack of data, the predictive value of simultaneous prelaryngeal and pretracheal lymph node metastasis could not be analyzed, which was speculated more valuable. As for the effect on the lateral lymph node metastasis, no study explored the association.

The existence incidence of prelaryngeal lymph node was 23–76%, and the metastatic incidence was 7.7–30.5% (16, 29, 31, 36). This might be the reason for the lower RRs. A previous meta-analysis suggested that the sensitivities of prelaryngeal lymph node metastasis in predicting contralateral paratracheal, central (without prelaryngeal), and lateral lymph node metastasis were not markedly high (9). The results of the present meta-analysis were consistent with the previous study. Therefore, the states of the combination of prelaryngeal and pretracheal lymph node metastasis might be a more effective prognostic factor and an indicator for paratracheal lymph node dissection. The American Thyroid Association Management Guidelines indicate that clinically involved central nodes are an intermediate risk factor and that central neck dissection should be performed in these patients (7). Similar to the clinically involved central nodes, the prelaryngeal and/or pretracheal lymph node metastasis confirmed by intraoperative frozen biopsy might suggest that central lymph node dissection is necessary and help to further distinguish the relative high-risk PTC from the low-risk PTC.

Substantial heterogeneity was observed among the studies concerning the relationship between pretracheal lymph node metastasis and ipsilateral paratracheal lymph node metastasis, and contralateral paratracheal lymph node metastasis, between prelaryngeal lymph node metastasis and central (without prelaryngeal) lymph node metastasis, and contralateral paratracheal lymph node metastasis, between pretracheal and/or prelaryngeal lymph node metastasis and ipsilateral paratracheal lymph node metastasis, and contralateral lymph node metastasis. Heterogeneity was the major problem that affected the reliability of the pooled effect size in the meta-analysis. The following factors might have influenced the heterogeneity (1): The stages of PTC varied among the included studies. Some studies included patients with PTC with the largest diameter exceeding 4 cm. It is well known that a larger diameter is a poor prognostic factor. Some studies also included patients with cN1 PTC, which already represented an intermediate risk (2). The lesion sites were not completely consistent. Not all studies only included patients with unilateral PTC, some studies included patients with bilateral PTC and/or isthmic PTC, which was a risk factor for prelaryngeal and/or pretracheal lymph node metastasis and then affected relationship and heterogeneity (3). The way to deal with data of patients without prelaryngeal lymph nodes was different among studies. These patients have not been included in analysis in some studies (4). Diverse surgical methods were performed, which might inaccurately determine the status of the lymph node (5). The characteristics of the populations varied in different studies (6). The confounding factors were different across these studies (7). The quality of studies (NOS scores) was not completely consistent.

There are several limitations to this meta-analysis. First, an analysis could not be performed in patients with unilateral pT1-2 PTC because of the lack of eligible data. Second, although we performed subgroup analysis, the result was always affected by some factors, such as bilateral lesions, isthmic lesions, and lack of information about the existence of prelaryngeal lymph nodes, and the heterogeneity did not always disappear. Third, there was a potential publication bias in studies that explored the relationship between pretracheal lymph node metastasis and contralateral paratracheal lymph node metastasis, and between prelaryngeal lymph node metastasis and contralateral paratracheal lymph node metastasis, even though they might be addressed by subgroup analysis. Fourth, studies that explored the influence of the combination of pretracheal and prelaryngeal lymph node metastasis are rare. Last, the exclusion of non-English-language studies might lead to bias.

## Conclusions

This meta-analysis demonstrated that both pretracheal lymph node metastasis and prelaryngeal lymph node metastasis were significantly associated with an increased possibility of both ipsilateral lymph node metastasis and contralateral paratracheal lymph node metastasis in PTC. A similar result was obtained between pretracheal and/or prelaryngeal lymph node metastasis and paratracheal lymph node metastasis. Moreover, prelaryngeal lymph node metastasis was positively correlated with the incidence of lateral lymph node metastasis. Considering the limited number of studies, it is necessary to conduct more studies that explore the association between the combination of pretracheal and prelaryngeal lymph node metastasis and paratracheal lymph node metastasis, as well as lateral lymph node metastasis in PTC, particularly unilateral pT1-2 PTC.

## Data availability statement

The original contributions presented in the study are included in the article/supplementary material. Further inquiries can be directed to the corresponding author.

## Author contributions

Study conception and design: BW, C-RZ, HL, X-MY, and JW. Acquisition of data: BW, C-RZ. Analysis and interpretation of data: BW, C-RZ, and HL. Drafting of manuscript: BW and C-RZ. Critical revision: BW, C-RZ, HL, X-MY, and JW. Final approval of the version to be submitted: BW, C-RZ, HL, X-MY, and JW. All authors contributed to the article and approved the submitted version.

## Funding

BW was supported by a nonprofit fund from CHINA HEALTH PROMOTION FOUNDATION. JW was supported by a grant from Scientific Research Fund of the Department of Science and Technology of Chengdu City (2015-HM01-00376-SF) and Science and Technology Program of Science & Technology Department of Sichuan Province (2015JY0190). The funding bodies had no role in the conception of the study, in the collection, analysis, and interpretation of data, in writing the manuscript and in the approval of the publication.

## Conflict of interest

The authors declare that the research was conducted in the absence of any commercial or financial relationships that could be construed as a potential conflict of interest.

## Publisher’s note

All claims expressed in this article are solely those of the authors and do not necessarily represent those of their affiliated organizations, or those of the publisher, the editors and the reviewers. Any product that may be evaluated in this article, or claim that may be made by its manufacturer, is not guaranteed or endorsed by the publisher.
